# Evaluation of Tamsulosin 0.4 mg versus 0.8 mg in management of lower urinary tract symptoms due to benign prostatic enlargement

**DOI:** 10.1007/s11255-023-03912-7

**Published:** 2024-01-14

**Authors:** Tarek Osman, Hossam Elawady, Khaled Fawaz, Mohamed Shabayek, Mohammed Darweesh Essam, Dana Osman, Karim Omar ElSaeed

**Affiliations:** 1 Urology Department, Ain Shams University, Cairo, Egypt; 2 Misr University for Science and Technology, Cairo, Egypt; 3 Faculty of Medicine, Ain Shams University, Cairo, Egypt

**Keywords:** Tamsulosin, Lower urinary tract symptoms, Benign prostatic enlargement, Maximum flow rate

## Abstract

**Purpose:**

To compare the efficacy and the safety of Tamsulosin 0.4 mg/day and 0.8 mg/day in patients suffering from lower urinary tract symptoms due to benign prostatic obstruction.

**Patients and Methods:**

A prospective interventional, double-blinded, controlled study was carried out on 93 patients who met the criteria and divided randomly into two groups: group A for Tamsulosin 0.4 mg/day and group B for Tamsulosin 0.8 mg/day. International prostate symptom score, post void residual urine volume, and maximum flow rate of urine were assessed before and after 4 weeks of treatment.

**Results:**

Both study groups showed a significant reduction in storage sub-score but only frequency was significantly reduced in group B (*P* < 0.001). On the other hand, Tamsulosin 0.8 mg was superior to Tamsulosin 0.4 mg regarding voiding sub-score except for straining (*P* = 0.325). Accordingly, the total international prostate symptom score was significantly improved in group B versus group A (*P* < 0.001). Furthermore, maximum flow rate and post-void residual urine volume were notably improved in Group B as compared to Group A (*P* < 0.001). Of all adverse events only dizziness was noted to be statistically significant in Group B versus Group A (*P* < 0.001).

**Conclusion:**

Tamsulosin 0.8 mg has shown better outcomes in treating patients who suffer from lower urinary tract symptoms due to benign prostatic enlargement than Tamsulosin 0.4 mg, and besides that, it is well tolerated.

**Trial registration number:**

M S 292/2020, SID: 373, date: 22/4/2020.

## Introduction

Benign prostatic enlargement (BPE) is the leading cause of lower urinary tract symptoms (LUTS) in elderly men. This condition is seen in 50% of men aged between 51 and 60 years, and more than 90% of men above 80 years old, increasing the need for efficient and enduring treatments. Management of BPE varies from watchful waiting to surgical intervention. The current medical therapies include α-adrenergic blockers (α-blockers), 5 α reductase inhibitors, Phosphodiesterase 5 enzyme inhibitors, and muscarinic receptor blockers (M3-blockers) [1].

Most physicians use α-blockers as the first line of treatment when treating patients with BPE-associated LUTS. The evidence that prostate smooth muscle contraction causes bladder outlet obstruction (BOO) justifies the use of α-blockers in treating BPE-associated LUTS. α1 adrenergic receptors have three subtypes: α1A, α1B, and α1D. 70% of human prostatic adrenoreceptors are made up of α1A which can reach 80% in BPE patients [2]^.^

Tamsulosin, a highly selective α1-blocker, lowers the tone of the smooth muscle contraction in the prostate, urethra, and bladder neck, reducing urine flow resistance [3]. It has more affinity for α1A receptors than for α1B receptors. That is why it has fewer cardiovascular adverse effects, and no interactions with antihypertensive medications [4].

Uroflowmetry parameters like maximum flow rate (*Q*_max_), average flow rate (*Q*_avg_), and post-void residual (PVR) urine volume, as well as International Prostate Symptom Score (IPSS), are used to evaluate the improvement in LUTS [5].

Compared to other α1-blockers, Tamsulosin causes fewer adverse effects such as dizziness, vertigo, first-dose syncope, and orthostatic hypotension. There was no statistically significant difference in blood pressure between Tamsulosin-treated, and Placebo-treated individuals according to the studies [6, 7]. On the other hand, Tamsulosin, frequently, causes delayed or retrograde ejaculation. This occurs by blocking the α1 adrenergic receptors in the vas deferens and the bladder neck, failing the internal sphincter to contract during ejaculation. Other less common adverse effects include headache, asthenia, and rhinitis-like symptoms which are likely to be brought on by suppression of serotonin’s release in the central nervous system [8].

In clinical experience, not all patients have reacted to Tamsulosin 0.4 mg once daily, necessitating the use of other therapies, or perhaps dose escalation. Tamsulosin 0.4 mg and 0.8 mg effects were compared in a small number of studies, mostly lacking blinded randomisation, or lack a control group. As a result, we aimed to evaluate the effectiveness of Tamsulosin 0.8 mg once daily compared to the traditional dose 0.4 mg, as well as the likelihood of any possible adverse events.

## Patients and methods

### Study design

This study was a double-blinded, randomized, prospective trial that was conducted from January 2020 to June 2021. A total of 211 patients from a single tertiary care facility were assessed for eligibility. Patients aged ≥ 50 and ≤ 90 years, who were diagnosed with BPE-associated LUTS and did not receive medical treatment for BPE in the last 2 weeks, were eligible. The exclusion criteria included a previous history of acute urinary retention (AUR) or prostate surgery, patients with chronic urinary retention, or prostate malignancy, and other causes of LUTS (urinary bladder stones, neurogenic bladder, or urethral stricture). 94 patients were excluded according to the exclusion criteria and 24 patients refused to participate. 93 patients were enrolled and consented to the study and its purpose. The study was approved by our institute’s ethical committee.

**Fig. 1:**
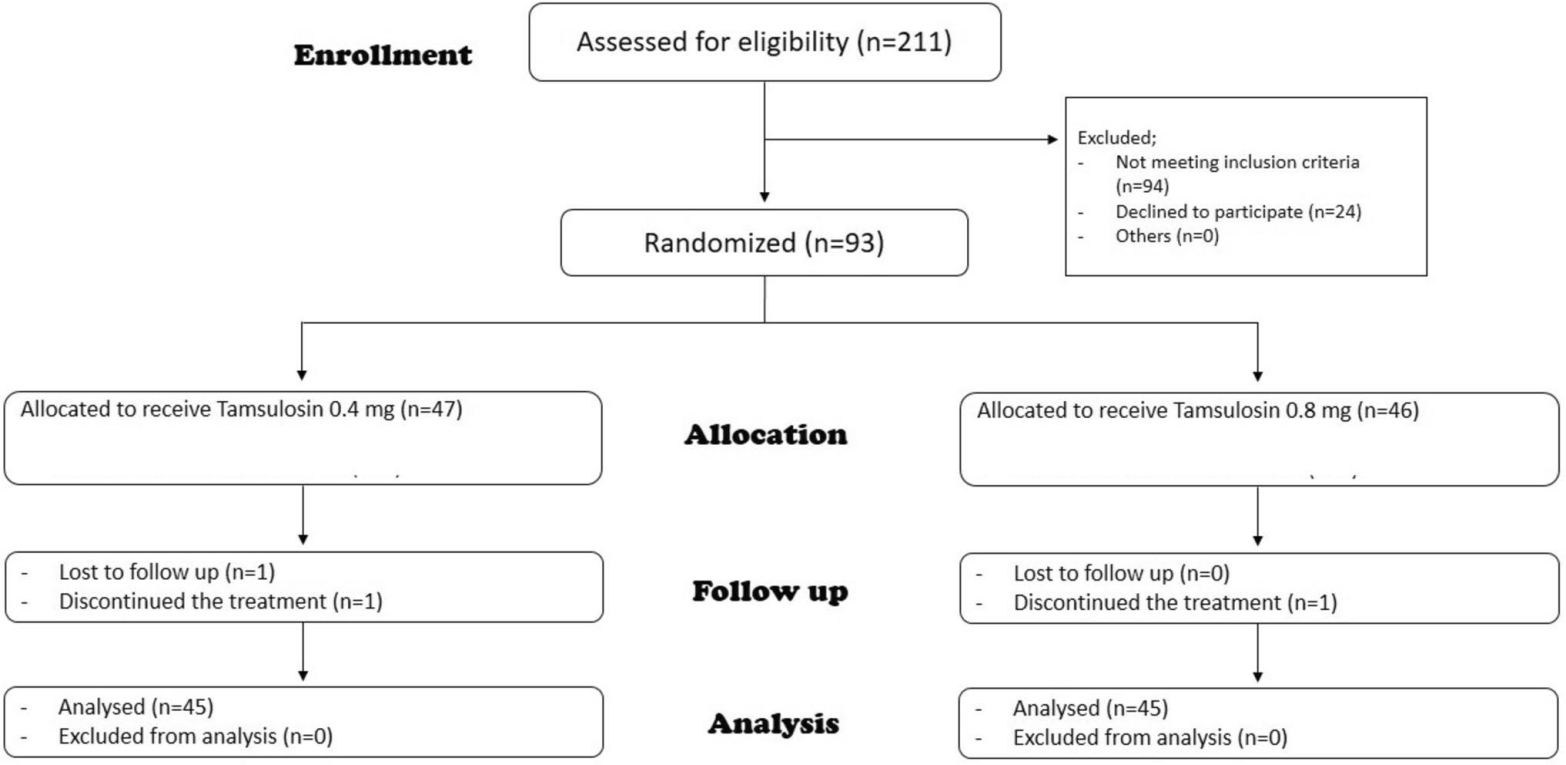
Flow diagram of the number of patients enrolled, randomized, and completing the study per treatment group

Before randomization, patients were evaluated by general history taking (smoking, lifestyle, past medical history, current medications, sexual life, and assessment of ejaculation activity), and physical examination (measurement of body mass index (BMI), systolic blood pressure (SBP), and diastolic blood pressure (DBP)). IPSS questionnaires were administered, and PVR urine volume and *Q*_max_ were evaluated by an abdominopelvic ultrasound and uroflowmetry respectively. Participants were randomized into two groups in a manner of 1:1 ratio (Group A received Tamsulosin 0.4 mg and Group B received Tamsulosin 0.8 mg). All study subjects entered the double-blinded phase by giving the investigator coded pill boxes to deliver to the participants. Each box contained 28 compartments for the 28 days of the study. We chose to perform a preliminary study for a short period of time (4 weeks) for two reasons. The first was justified by the fact that most side effects of alpha blockers tend to express themselves in the initial doses. Secondly, we had concerns that subjects may not exhibit compliance with the drug under trial if the study duration was prolonged, especially Egyptian patients have a reputation of being non-compliant. So, to avoid a big segment of the patients aborting the trial, we chose to start with 4 weeks. In the event that the bigger dose proved its potency and safety, a second study would be designed on a longer scale. Each compartment had either 2 tablets of Tamsulosin 0.4 mg (for group B) or a tablet of Tamsulosin 0.4 mg and a placebo one with inactive ingredients (for group A). Both tablets were taken together as one dose. After 4 weeks of treatment, patients were re-evaluated by IPSS questionnaire, measurement of *Q*_max_, PVR urine volume, SBP, and DBP, and asking about headache, dizziness, and ejaculation abnormality. There were 3 patients who dropped out of the study, 2 of them were due to adverse events (dizziness), and one failed to continue the study. Figure 1 demonstrates our consort flowchart.

### Study assessment

Efficacy was determined by assessment of the primary endpoints, which were the changes in IPSS, PVR urine volume, and *Q*_max_ before and after the treatment. Regarding secondary endpoints, safety was assessed by summarizing the incidence of adverse effects and measurement of SBP and DBP.

### Statistical analysis

Based on the postulated improvement of 50% of cases in Tamsulosin 0.4 mg compared to that of 80% of cases in Tamsulosin 0.8 mg, the alpha error is 5% and the power of the study is 80%. Therefore, the required sample size is 90 patients, 45 in each group. The program for sample size calculation is STATA 10.

The collected data were coded, tabulated, and statistically analyzed using IBM SPSS statistics (Statistical Package for Social Sciences) software version 22.0, IBM Corp., Chicago, USA, 2013. Quantitative normally distributed data was described as mean ± SD (standard deviation) after testing for normality using the Shapiro–Wilk test, then compared using independent *t* test (two independent groups) and paired *t* test (paired data). Qualitative data were described as numbers and percentages and compared using the Chi-square test. A *P* value < 0.050 was significant, otherwise was non-significant.

## Results

### Patients’ demographics and baseline characteristics

A total of 93 patients were randomized to Tamsulosin 0.4 mg (group *A* = 47), and Tamsulosin 0.8 mg (group *B* = 46). Regarding demographic characteristics, there was no significant difference between both groups as summarized in Table 1. Before starting the treatment, there were no significant differences between the 2 groups regarding IPSS, PVR urine volume, or *Q*_max_.

**Table 1:** Mean change ± SD of patients’ demographics

	Tamsulosin 0.4 mg	Tamsulosin 0.8 mg	*P* value*
Age	64.9 ± 6.6	63.8 ± 5.9	0.399
BMI	25.9 ± 1.9	26.2 ± 1.8	0.436
SBP	132.9 ± 12.0	135.2 ± 11.6	0.350
DBP	81.6 ± 8.7	81.4 ± 9.5	0.916
Prostate Size	50.4 ± 7.3	49.1 ± 7.3	0.372

^*^Independent *t* test (comparison between groups)

### Efficacy

**Fig. 2:**
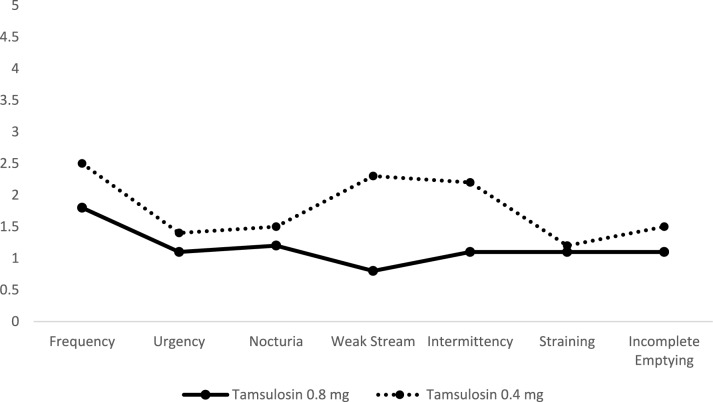
The difference between both groups at follow-up in IPSS sub-scores

A statistically significant improvement in Total IPSS scores was observed from baseline (29.4 ± 2.6, severe) to the follow-up visit (8.5 ± 1.7, mild) in patients who received Tamsulosin 0.8 mg (*P* < 0.001). This improvement was seen in frequency, weak stream, intermittency, and incomplete emptying. On the other hand, no significant changes were noted between both groups for urgency, nocturia, or straining. *Q*_max_ was significantly greater in group *B* than in group *A* (*P* < 0.001). The mean change in *Q*_max_ was 6.1 ± 1.2 ml/s, and 1.9 ± 0.5 ml/s. for group *B*, and group *A* respectively. Furthermore, there was a significant reduction in PVR urine volume in group *B*. The mean change in group *B* was − 36 ± 6.5 ml. in comparison to that of group *A* (− 28 ± 6.8 ml., *P* < 0.001). These changes are summarized in Table 2 and Fig. 2.

**Table 2:** Mean change ± SD from baseline to follow-up in primary efficacy parameters

	Tamsulosin 0.8 mg	Tamsulosin 0.4 mg	*P* value**
Total IPSS			
Baseline	29.4 ± 2.6	29.1 ± 1.3	0.577
Follow up	8.5 ± 1.7	12.4 ± 1.0	< 0.001*
Change	− 20.8 ± 1.7	− 16.8 ± 1.7	< 0.001*
*Q*_max_			
Baseline	8.8 ± 1.5	8.4 ± 1.1	0.154
Follow up	14.9 ± 2.0	10.4 ± 1.3	< 0.001*
Change	6.1 ± 1.2	1.9 ± 0.5	< 0.001*
PVR			
Baseline	59.7 ± 7.1	61.5 ± 8.2	0.281
Follow up	23.6 ± 4.3	32.9 ± 7.0	< 0.001*
Change	− 36.1 ± 6.5	− 28.6 ± 6.8	< 0.001*

^*^Significant

^**^Independent *t* test (comparison between groups)

### Safety

**Fig. 3:**
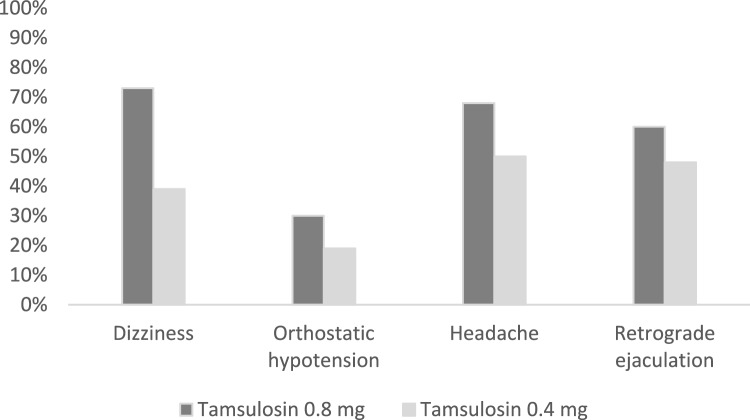
Percentage of patients in both groups who experienced adverse events

Dizziness was statistically more frequent in group *B* (73%) than in group *A* (39%) (*P* < 0.001). Retrograde ejaculation was also a frequent adverse event in both groups, especially in group B (60%). Despite that both groups had no significant differences (*P* = 0.290). Orthostatic hypotension occurred by 30% in group B and 19% in group A without significant difference (*P* = 0.227). Also, both groups reported drug-related headaches but without a statistically significant difference (*P* = 0.085). Adverse events are summarized in Fig. 3.

## Discussion

This was a prospective, randomized, double-blind study, conducted on 90 patients who had BPE-associated LUTS.

In this study, the total IPSS score improved significantly with group B who received Tamsulosin 0.8 mg (*P* < 0.001), without noted changes between the 2 groups in straining, nocturia, or urgency. In a phase 3 multicenter placebo-controlled study [9], patients with BPH were randomized to receive Tamsulosin 0.8 mg, Tamsulosin 0.4 mg, and placebo. The mean change in IPSS was significantly greater in both Tamsulosin groups than that of placebo (*P* < 0.001) with the superiority of Tamsulosin 0.8 mg over Tamsulosin 0.4 mg in voiding sub-scores (*P* = 0.007). Another study done on 81 Taiwanese patients who were dissatisfied with the usual dose of Tamsulosin (0.2 mg, due to the lower BMI in Asian people) and asked to escalate the dose to 0.4 mg, found a significant improvement in total IPSS from baseline (14.94 ± 7.41) to the end of 12-week period (7.36 ± 5.77, *P* < 0.001) [10]. The results of these 2 studies were in agreement with ours. In contrast, no statistically significant difference was noted in the mean change in IPSS from the baseline to the endpoint between Tamsulosin 0.4 mg and Tamsulosin 0.8 mg (− 5.09 ± 0.41 and − 5.76 ± 0.41, respectively) in another study [11]. In a multicenter double-blind study for 12 weeks of treatment with a placebo, Tamsulosin modified release (MR) 0.4 mg, Tamsulosin oral-controlled absorption system (OCAS) 0.4 mg and Tamsulosin OCAS 0.8 mg, no statistically significant change was found in IPSS between Tamsulosin MR 0.4 mg and Tamsulosin OCAS 0.8 mg (*P* = 0.999) [12].

Regarding *Q*_max_, we found a statistically significant improvement in both groups relative to the baseline. The mean change in *Q*_max_ was significantly higher in group *B* (6.1 ± 1.2 ml/s and 1.9 ± 0.5 ml/s for group *B* and *A* respectively, *P* < 0.001). Two studies [10, 13] compared the effect of Tamsulosin 0.4 mg versus Tamsulosin 0.2 mg, and found a significant improvement in *Q*_max_ for Tamsulosin 0.4 mg. In one study [10], *Q*_max_ increased significantly from baseline (11.37 ± 6.04 ml/s) to Week 12 (13.06 ± 6.18 ml/s) (*P* = 0.0037). In the other study [13], the mean change in *Q*_max_ from the baseline to Week 12 for Tamsulosin 0.2 was − 0.25 ± 0.3 ml/s and 3.0 ± 0.48 ml/s for Tamsulosin 0.4 mg (*P* < 0.001). The results from the fore mentioned studies were in agreement with ours that doubling the dose of Tamsulosin had better outcomes. On the other hand, another study [9], found no significant difference in the mean change between Tamsulosin 0.4 mg and 0.8 mg (1.75 ± 3.5 and 1.78 ± 3.3 ml/s, respectively). And in yet another study [11], although the results in patients treated with Tamsulosin were significant in comparison to placebo (P < 0.05), there was no significant difference between Tamsulosin 0.4 mg and 0.8 mg.

Our study found that the reduction in PVR urine volume was more significant in group *B* (− 36.1 ± 6.5 ml) than in group *A* (− 28.6 ± 6.8 ml) (*P* < 0.001). Unlike our study, others found no significant change in PVR urine volume when the dose of Tamsulosin was upscaled from 0.2 mg to 0.4 mg (*P* = 0.5486) [10]. Another study also found no significant change between Tamsulosin 0.2 mg and 0.4 mg [13].

Overall, Tamsulosin was well tolerated at doses of 0.4 mg and 0.8 mg. The incidence of adverse events like headache, abnormal ejaculation, and orthostatic hypotension was more frequent with Tamsulosin 0.8 mg but not significant. Only dizziness was significantly more frequent in group B (73%, *n* = 33) than in group A (39%, *n* = 18, *P* < 0.001). On the other hand, abnormal ejaculation was significantly frequent with Tamsulosin 0.8 mg in some studies [9, 11, 12].

## Conclusion

Treating patients who have symptomatic BPE and complain of severe LUTS with Tamsulosin 0.8 mg once daily is more effective than Tamsulosin 0.4 mg with significant improvement in IPSS, *Q*_max_, and PVR urine volume. Tamsulosin 0.8 mg is well tolerated showing no significant difference from Tamsulosin 0.4 mg. Consequently, we do believe it is safe to increase the dose to 0.8 mg according to the severity of the symptoms without increasing the incidence of adverse events.

## Limitations of the study

Absence of Tamsulosin 0.8 mg as one tablet in our country was one of the obstacles that was solved by giving the patients 2 tablets of Tamsulosin 0.4 mg. In addition, other adverse events might not have been detected due to the short-term period of the study like impacts on sexual function. Furthermore, will the drug effect decline with time, is a question yet to be answered. We therefore recommend a second trial studying more subjects for a longer duration before any solid recommendations on the role of a double dose tamsulosin could be made.

## Data Availability

Data availability applicable upon reasonable request.

## Abbreviations

LUTS: Lower urinary tract symptoms
BPE: Benign prostatic enlargement
BOO: Bladder outlet obstruction
*Q*_max_: Maximum urinary flow rate
*Q*_avg_: Average urinary flow rate
PVR: Post-void residual
IPSS: International prostate symptom score
QoL: Quality of life
AUR: Acute urinary retention
BMI: Body mass index
SBP: Systolic blood pressure
DBP: Diastolic blood pressure
MR: Modified release
OCAS: Oral-controlled absorption system

## Declarations

### Human and animal participants

This research involves human participants.
